# Lipid Storage Myopathy in Behçet's Disease: A Rare Cause of Elevated Serum Creatine Kinases Levels

**DOI:** 10.1155/2012/283259

**Published:** 2012-06-28

**Authors:** Sedat Yilmaz, Muhammet Cinar, Yıldırım Karslioglu, Ismail Simsek, Hakan Erdem, Salih Pay, Ayhan Dinc

**Affiliations:** ^1^Division of Rheumatology, Gulhane School of Medicine, Ankara, 06018 Etlik, Turkey; ^2^Department of Pathology, Gulhane School of Medicine, Ankara, 06018 Etlik, Turkey

## Abstract

Muscular involvement in Behçet's disease is rare and there are only a few case reports in the literature. The causes of elevated muscle enzymes in a patient with Behcet's disease are many, including myositis, drug-induced myopathy, metabolic myopathy, and the disease itself. We herein have defined an algorithmic approach to a patient with Behcet's disease and elevated muscle enzymes and report a case of coexisting of lipid storage myopathy.

## 1. Introduction

Behçet's disease (BD) is a systemic vasculitis characterized by relapsing episodes of oral aphthous ulcers, genital ulcers, skin lesions, and ocular lesions. Other systems including vascular, gastrointestinal, and neurological systems can be affected; BD is more frequent and has more serious clinical course in men [1]. Muscular involvement is rare and there are only a few case reports in the literature [2–4].

Disorders of lipid metabolism, namely, “lipidoses” or “lipid storage myopathies,” can cause variable clinical presentations, often-involving skeletal muscle. Although other systems, including cardiac, hepatic, and metabolic, can be involved, most frequent complaints are muscle pain and fatigue, usually induced by exercise, fasting, and infections [5]. Diagnosis is important, since they can cause morbidity and mortality and are potentially treatable conditions. Blood tests (serum creatin kinase (CK), lactate, acylcarnitine profile, and amino acids), urine testing (organic acids and myoglobin), muscle biopsy (light and electron microscopy and biochemical assays), electromyography (EMG), exercise testing, genetic testing, and magnetic resonance spectroscopy (MRS) are helpful in establishing the diagnosis.

## 2. Case

**Figure 1:**
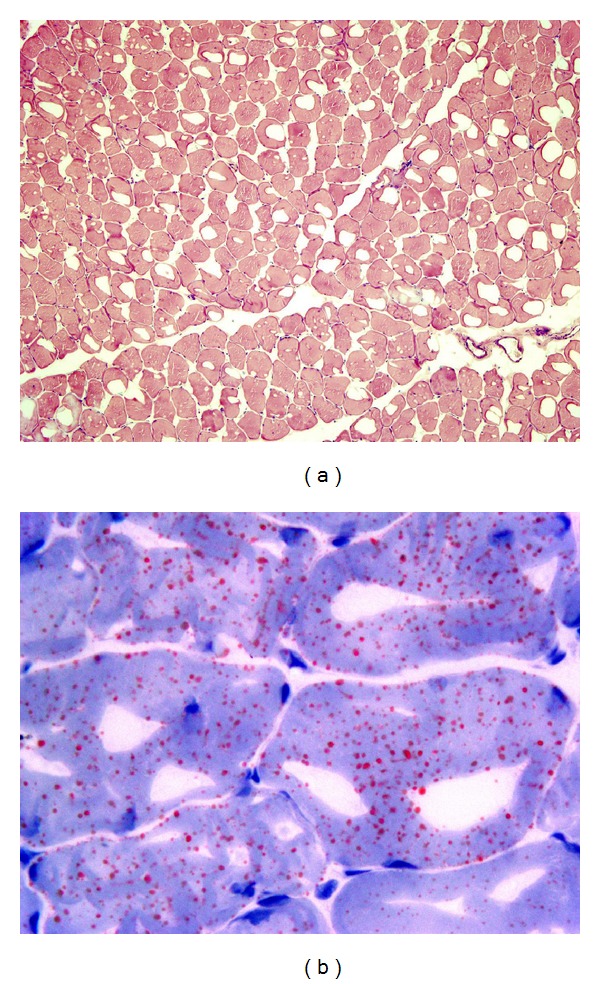
(a) H&E stained frozen section of the muscle biopsy showing normal architecture (×100). (b) Oil Red-O method, which is used for highlighting neutral triglycerides and lipids in frozen sections, showed several orange-red-stained droplets of lipids, a finding consistent with the clinically suspected diagnosis of lipid storage disease (×1000).

A 19-year-old man presented with oral aphthous ulcers, genital ulcers, erythema nodosum, and myalgia on lower extremities. He had been diagnosed as BD and was using colchicine 0.6 mg three times a day for approximately seven years. Physical examination was revealed two minor oral ulcers, two active genital ulcers and four cicatrices in scrotum and an erythema nodosum in anterior crural region, and he has low-grade fever (37.6°C). In neurological examination, muscle strength was normal in all extremities. The initial laboratory examination showed that erythrocyte sedimentation rate and C-reactive protein levels were high (22 mm/h and 8.2 mg/L, resp.) and muscle enzymes including creatine phosphokinase (CPK), lactate dehydrogenase (LDH), and aspartate aminotransferase (AST) levels were also elevated (1746 U/L, 480 U/L, and 58 U/L, resp.). Elevated muscle enzymes were attributed to the fever accompanying to active disease. So, to control the disease activity and to provide symptomatic relief, we started Prednisolon (0.5 mg/kg). In the fourth day of the treatment, all his complaints were alleviated and muscle enzymes began to decrease (CPK, 804 U/L; LDH, 321 U/L). Prednisolon treatment was tapered quickly to discontinue within 2 weeks as of the beginning of the therapy. As the disease was limited to mucocutaneous regions, we decided to continue colchicine treatment and acknowledge him to come to control visit one month later. At the second admission, he had no active lesions including oral and genital ulcers and erythema nodosum. In laboratory evaluation, acute phase reactants were normal but muscle enzymes were still high (CPK, 6902; LDH, 503 U/L). So, we performed electromyography, which reveals nonspecific changes including short-duration polyphasic MUAPs. Therefore, elevated muscle enzymes was considered to be an adverse effect of colchicine, namely colchicine induced myopathy. So, we decided to discontinue this medication and began azathioprine 50 mg, twice daily, and scheduled a visit 4 weeks later. At the time of last visit, his muscle enzymes were still elevated. So, we decided to perform a muscle biopsy. His only complaint was widespread mild myalgia, which was more prominent in lower extremities and occurring especially after exercise. In muscle biopsy, the overall morphology and the architecture with hematoxylin and eosin (H&E) stain were within normal histological appearance (Figure 1(b)). However, Oil Red-O staining disclosed several orange-red-stained droplets of lipids in the sarcoplasms of many muscle fibers, which was consistent with lipid storage myopathy (Figure 1(a)).

## 3. Discussion

Behçet's disease is a systemic vasculitis with an unknown etiology. Its prevalence is low except the ancient “silk road” countries including Turkey, Iran, and Japan. Although the most frequently encountered signs and symptoms of disease are mucocutaneous lesions, ocular, vascular, gastrointestinal, and neurological involvements can also occur. On the other hand, muscle disease associated with BD is very rare and reported as case reports [6]. Most of the patients presented with localized myositis; however, three patients had generalized symptoms [7].

In our case, the patient's presenting symptoms were oral, genital ulcers, and erythema nodosum. Elevated muscle enzymes were incidental findings and firstly considered to be due to fever secondary to the active disease. Later on, since serum levels of the muscle enzymes continued to be high, we discontinued his colchicine treatment, because of the suspicion of colchicine-induced myopathy. Despite cessation of the drug, muscle enzymes kept on elevating. So we decided to perform muscle biopsy and diagnosis of lipid storage myopathy was made. Our patient's symptoms regarding with the lipid storage myopathy were very subtle and this delayed the diagnosis.

To our knowledge, it is the first report of BD coexisting with lipid storage myopathy, we wanted to report and call attention to the cases of elevated muscle enzymes in BD. In conclusion, if elevated muscle enzymes were found in a patient with BD, the probability of the other causes independent from BD should be keep in mind, along with the drug-and disease-related etiologies.
